# Impact on patient-provider relationship and documentation practices when mental health patients access their electronic health records online: a qualitative study among health professionals in an outpatient setting

**DOI:** 10.1186/s12888-022-04123-7

**Published:** 2022-07-28

**Authors:** Paolo Zanaboni, Eli Kristiansen, Ove Lintvedt, Rolf Wynn, Monika A. Johansen, Tove Sørensen, Asbjørn J. Fagerlund

**Affiliations:** 1 Norwegian Centre for E-Health Research, University Hospital of North Norway, Tromsø, Norway; 2 Department of Clinical Medicine, UiT The Arctic University of Norway, Tromsø, Norway; 3 Helse Nord IKT HF, Tromsø, Norway

**Keywords:** Electronic health record, Patient accessible electronic health records, e-health, Mental health, Patient empowerment

## Abstract

**Background:**

Patient accessible electronic health records (PAEHR) hold the potential to increase patient empowerment, especially for patients with complex, long-term or chronic conditions. However, evidence of its benefits for patients who undergo mental health treatment is unclear and inconsistent, and several concerns towards use of PAEHR emerged among health professionals. This study aimed at exploring the impact of PAEHR among mental health professionals in terms of patient-provider relationship, changes in the way of writing in the electronic health records and reasons for denying access to information.

**Methods:**

In-depth qualitative interviews with health professionals working in two mental health outpatient clinics at Helgelandssykehuset in Northern Norway, one of the first hospitals in Norway to implement the PAEHR in 2015. The interviews were conducted by phone or videoconferencing, audio recorded and transcribed verbatim. Data were analyzed by a multidisciplinary research team using the Framework Method.

**Results:**

A total of 16 in-depth qualitative interviews were conducted in April and May 2020. The PAEHR implemented in Norway was seen as a tool to increase transparency and improve the patient-provider relationship. The PAEHR was seen to have negative consequences only in limited situations, such as for patients with severe mental conditions, for child protective services when parents access their children’s journal, or for patients with abusive partners. The functionality to deny access to the journal was used rarely. A more common practice for making information not immediately available was to delay the final approval of the notes. The documentation practices changed over the years, but it was not clear to what extent the changes were attributable to the introduction of the PAEHR. Health professionals write their notes keeping in mind that patients might read them, and they try to avoid unclear language, information about third parties, and hypotheses that might create confusion.

**Conclusions:**

The concerns voiced by mental health professionals regarding the impact of the PAEHR on the patient-provider relationship and practices to deny access to information were not supported by the results of this study. Future research should explore changes in documentation practices by analysing the content of the electronic health records.

## Background

### Patient-accessible electronic health records

Providers and policymakers are pursuing strategies to increase patient empowerment [1]. One way to increase patient empowerment might be to give patients access to their health records. Fifty years ago it was predicted that giving patients “complete and unexpurgated copy of all medical records” would enhance patient autonomy, improve patient-provider relationships and serve as educational tool [2]. Today, technologies are in place to allow patients online access to their health records.

An electronic health record (EHR) is the electronic collection of clinical data, and can include clinical assessments, nursing documentation, laboratory and radiology results, medication and allergy information and discharge letters [3]. Patient accessible electronic health records (PAEHR) [4] are online services providing patients the ability to view their EHR [5], collect information before a visit, schedule appointments, renew prescriptions, ask a question and receive reminders [6].

Online access to EHR holds the potential for improved communication between patients and providers [7, 8]. The PAEHR can enhance the provision of patient-centered care [9–11], making it easier for most people to understand their health status and increase adherence [12]. This can, in turn, lead to improved clinical outcomes [3], thus enabling patients to more effectively self-manage their health conditions and actively participate in consultations [5, 13–15].

The EHR is one of the main tools for multi-professional cooperation and communication with patients. Furthermore, the EHR should be appreciated as an agent, playing an active and constitutive role in setting objectives, planning, documenting delivery of care and assessing outcomes [16]. The content of the EHR is important as it serves professionals in making decisions and documenting the deliberations which have been made and the care that has been given [16].

### Online access to EHR in Norway

All citizens and residents in Norway have the right to read the information in their health records which have been created by a health care provider [17]. According to the Norwegian Patients’ Rights Act, a patient may be denied access if this is absolutely necessary to avoid endangering the patient’s life or serious damage to the patient’s health, or if access is clearly inadvisable because it may cause harm to persons close to the patient.

The EHR is fully established by all Norwegian hospitals. The national health portal helsenorge.no was established in 2011 to accommodate digital patient services after secure login [18]. In 2012, the Ministry of Health and Care Services published a governmental white paper which stated that patients should have online access to their EHR [19], and the work with establishing the PAEHR as a service at helsenorge.no started. To date, hospitals in three of the four health regions in Norway (Northern Norway since 2015, Western Norway since 2017 and South-Eastern Norway since 2019) provide the service through helsenorge.no, with few variation across regions.

In general, all documents available in digital format, included psychiatry reports, are made available to the patient after they are approved by health professionals, unless health professionals decide to deny access. Use of the PAEHR service is not mandatory. The EHR consists of many different types of documents, some of which have been manually scanned.

### Online access to EHR in mental health

While many adults want full access to their EHR [15] and report predominantly positive experiences with the PAEHR [20], there is a clear predominance of fears and concerns among mental health professionals regarding the PAEHR, including an increased clinical burden owing to more documentation efforts [20]. Professionals are also worried about possible harm triggered by reading the notes [20] which, in turn could impact negatively on the patient-provider relationship [5]. One common example of concern is related to patients with severe mental illness reading their EHR online. Professionals in mental health care have expressed concerns that the transparency of PAEHR may cause “*unnecessary worry, confusion, or distress”* among patients who read their mental health progress notes without guidance from their clinicians [21], thus damaging the patient-provider relationship [11, 22]. In a brief survey of the Veterans Health Administration mental health clinicians’ experiences with the OpenNotes Movement, approximately half of the clinicians did not feel that mental health OpenNotes was a good idea [23]. In particular, they expressed concern over potential negative consequences and reported “*making changes to their note writing practices*”, including writing fewer details, changing the tone of the note, and writing less information about diagnoses. Moreover, up to 36% of doctors reported changing documentation content, and up to 21% reported “*taking more time writing notes”* [8]. Mental health clinicians in the United States also claimed that they were “*more careful”* about what they wrote to protect themselves and their patients [21].

In Sweden, access by patients to their EHR was first introduced in a pilot county since 2012 and then extended to several regions. This service was considered controversial and criticism arose from the clinical professions, mainly physicians. In particular, a number of health professionals have been reluctant to open for access to psychiatric records, considering it too sensitive [24]. Another study from Sweden revealed changes in documentation practices: over 60% of psychologists and nearly 40% of doctors stated that they were “*less candid in their documentation”*, which could in turn negatively influence both the work of the health professionals and the overall aim of having more informed and active patients [25]. Moreover, approximately one-fourth of health professionals believed that patients found the notes *“more confusing than helpful”*, and 33.5% thought that these patients worried more [26].

In Norway, patients who undergo mental health treatment have access to their EHR. In a survey conducted in 2016, patients reported that online access to EHR helped them gain a better understanding of their health status and follow up their treatment more closely [27]. Patients also appreciated the possibility to read easily all the information that health professionals wrote about them after attending visits, thus becoming more confident in understanding it, reporting mistakes or misunderstandings, and be better prepared for future visits. This was particularly important for patients with chronic conditions, as well as for patients who underwent mental health treatment. At the same time, health professionals expressed a number of concerns about online access to EHR for mental health patients, including how communication and the patient-provider relationship changed [28].

Another survey conducted in 2016 with 457 health professionals also revealed some challenges with the service [29]. Major differences in experience and attitude were found between psychiatric and somatic care. Overall, 43.9% of the health professionals in psychiatry reported that they *changed the way they wrote in the EHR* after the service was established (compared to 23.6% in somatic care). Moreover, 60% of the health professionals in psychiatry (compared to 15.2% in somatic care) discussed with a colleague whether to deny a patient access to information in their EHR. Some respondents thought that PAEHR was *not suitable for the sickest and most vulnerable patients*, such as those suffering from schizophrenia, bipolar disorder as well as those with severe depression/suicidality. Respondents commented that patients with severe illness might misunderstand information, especially in the middle of a therapy period, and refuse to speak with health professionals based on what they had read in the journal. A number of health professionals *denied access to information* that they worried might harm the patient or their relationship with the patient. Some also pointed out that the functionality for denying access to information was complicated to use. Other respondents reported that they omitted some information from the EHR or *wrote a “hidden” journal* containing off-the-record information they did not want the patient to read. Others delayed approval of some notes until after the therapy period was completed. A number of respondents felt that they had to spend more time to evaluate what to write. If they decided to deny access to information, this could harm the patient-provider relationship, as the patient might become suspicious and mistrustful.

### Study aim

The PAEHR in Norway was created primarily as an offer to all patients, regardless of their clinical and psychological status. While the PAEHR seems to have a positive impact on patient empowerment, especially for patients with complex, long-term or chronic conditions, evidence of its benefits for patients who undergo mental health treatment is still unclear and inconsistent. Several concerns have emerged among health professionals. The current study aimed at exploring the impact of PAEHR among mental health professionals. In particular, three research questions are addressed in this study: 1) How does the PAEHR impact on the patient-provider relationship? How does the PAEHR impact on the way of writing in the EHR, 3) How does the PAEHR impact on practices to deny access to information?

## Methods

### Data collection

We conducted a qualitative study among health professionals in an outpatient setting. A qualitative approach was chosen due to its ability to gather in-depth information and detailed experiences from users and its suitability to address the research questions. In-depth qualitative interviews were conducted with health professionals working with patients in mental health services. A semi-structured interview guide was used to explore the impact of PAEHR among mental health professionals in terms of patient-provider relationship, changes in the way of writing in the electronic health records and reasons for denying access to information. The interview guide was developed by the Norwegian Centre for E-health Research, Helgelandssykehuset, UiT The Arctic University of Norway, The Northern Norway Regional Health Authority and the patient organization Mental Helse. Some questions were also based upon qualitative feedback provided in past surveys.

Interviewees were health professionals working in two mental health outpatient clinics at Helgelandssykehuset in Northern Norway, one of the first hospitals in Norway to implement the PAEHR in 2015. Previous research showed that there were significant differences among health professionals in the way the PAEHR impacts on clinical practice and working processes [28, 29]. As a consequence, different health professionals (e.g. doctors, nurses, psychologists, physiotherapists and social workers) were involved. Representatives from the research group presented the background for the project to the clinicians at two locations (Brønnøysund and Sandnessjøen) followed by an invitation to participate in the interview.

One interviewer (OL), who did not have any relationship with the interviewees beforehand, conducted the interviews by phone or videoconferencing in April and May 2020. The interviewer presented topics using the interview guide, facilitated the discussion and followed up with further questions. The interviewees could discuss their experiences freely. Interviews were conducted until data saturation was reached. Interviews were audio recorded and transcribed verbatim.

### Data analysis

Data were analyzed by a multidisciplinary team consisting of three members (PZ, EK and AJF). Qualitative data collected from the interviews were analyzed using the Framework Method [30]. The Framework Method was chosen due to its suitability for multi-disciplinary health research teams, which is common in disciplines familiar with qualitative research, psychiatry [30]. The procedure for analysis consists in the following steps: 1) transcription, 2) familiarization with the interview, 3) coding, 4) developing a working analytical framework, 5) applying the analytical framework, 6) charting data into the framework matrix, 7) interpreting the data. Several iterations of the analytical framework are often required before no additional codes emerge. After transcription of the audio recordings, a sample of two interviews was randomly selected to let the research team familiarize itself with the transcripts and develop initial impressions and potential ideas for codes. Transcripts were then thoroughly read and independently analyzed by each member of the team. Interesting segments of text were underlined and notes made in the margins of the transcripts to describe the content of each passage with coding labels, as well as with more detailed information supporting the interpretation of the results. The team met to share the coding labels assigned to the transcripts from the two interviews. A working analytical framework was developed.

The remaining transcripts were then assigned to the members of the research team and analyzed using the analytical framework. New codes which were not included in the analytical framework were assigned as additional topics emerged. Regular team meetings were conducted to discuss new codes, group together codes which were conceptually related, and refine the analytical framework. The final analytical framework was applied to all the transcripts by assigning appropriate codes to each meaningful passage of text. Data were summarized in a framework matrix consisting of one column per interviewee and one row per code. Data from transcripts were inserted into the corresponding cell of the framework matrix and reviewed to make connections across interviewees and categories and to identify common themes as well as individual differences. The Standards for Reporting Qualitative Research (SRQR) was used to report the results from this study [31].

## Results

### Analytical framework

The final analytical framework (Table 1) consisted of eighteen codes grouped into four categories, each including a brief explanatory description of their meaning.

**Table 1:** Analytical framework

CATEGORIES AND CODES	DESCRIPTION
**General thoughts on the service**	
Inform patients about the service	Whether health professionals tend to inform patients
Scope and use	How much the service is used and who uses it
Training on the service for health personnel	Whether health professionals attended courses
Internal routines and practices	Formal or informal practices (e.g. discussions with colleagues)
**Patient-provider relationship**	
Transparency	The content of the EHR is visible to patients
Unsuitability	Whether the service is unsuitable to some patients
Relationship with the patient	How the service affects the relationship with patients
Roles of caregivers, children and third parties	How caregivers affect use of the service
EHR can be used as legal document	PAEHR as a service to patients vs EHR as a legal document
Use of PAEHR in treatment	Whether the service is actively used in patient treatment
**Way of writing in the EHR**	
Changes in writing	E.g. writing shorter sentences, less use of medical words
Changes in workflow	Whether the service resulted in changes in work practices
Consequences for the EHR as a work tool	Whether the service affected the main role of the EHR
**Practices to deny access to information**	
Knowledge of the functionality	Whether health professionals are aware of the functionality
Use of the functionality	How much and when the functionality is used
Reflections around the functionality	What health professionals think about the functionality
Avoid to write in the EHR	Whether omitting information in the EHR is applied
Other methods of making information not accessible	E.g. “hidden” or “shadow” journal

### Characteristics of the interviewees

A total of 16 in-depth qualitative interviews were conducted with health professionals working in two mental health outpatient clinics at Helgelandssykehuset. Each interview lasted approximately from 30 to 60 min. There were 14 women (87.5%) and 2 men (12.5%) and their age distributed as follows: 4 respondents aged < 30 years, 1 respondent aged 30–39 years, 2 respondents aged 40–49 years, 8 respondents aged 50–59 years, and 1 respondent aged > 60 years. Interviewees had the following educational backgrounds: 3 psychologists, 2 psychology students in their last year, 1 with bachelor in psychology, 4 nurses, 2 social educators, 1 social worker, 2 psychiatrists and 1 doctor undertaking specialization. Ten of the respondents (62.5%) had worked at the mental health outpatient clinics at Helgelandssykehuset since before the implementation of the PAEHR in 2015.

### General thoughts on PAEHR

#### Inform patients about the service

Most of the respondents tend to inform their patients about the PAEHR. Some health professionals are used to inform the patients about the PAEHR at the very first appointment, and even encourage them to use the service actively and read their journal before the next appointment. This is seen as especially important for patients with a tendency towards suspicion.

« I started actively asking my patients to read their records regularly» [# 13]

Other health professionals do that less systematically, and sometimes forget to inform the patients.

«Yes, I usually do that. But there isn’t any form of control […] but it usually happens during the first consultations» [#7]

Those who are not used to actively inform their patients mentioned that patients automatically receive information about the service in a standard notice letter from the hospital.

#### Scope and use

Overall, many interviewees had the impression that patients do not seem so interested in using the PAERH and, as a consequence, most of the patients do not use the service very actively, unless explicitly discussed in consultations.

«I think that, during the years I have worked here, maybe four patients have accessed actively their records and read them regularly» [#10]

Despite the PAEHR was introduced in 2015, one respondent mentioned that the service might be still perceived as something relatively new, and its use can therefore expected to increase in the future.

«The service will be further developed so that it will become more transparent since patients will have access from their mobile phones, and I think this will change their habits» [#13]

#### Training on the service for health personnel

Health professionals working at the two clinics received general training on the EHR via an Internet-based learning module in the past, but their memories were rather vague. Some interviewees also mentioned that, during their professional education, it was emphasized that the journal is the patient’s property. It was also pointed out the need for more training in writing in the EHR.

«During our studies we learned to a large extent that the health record is basically patient’s property, so it is always something that they can read and that can be discussed» [#6]

However, interviewees received little formal training on the PAEHR, and the topic was mostly limited to discussions with leaders and supervisors.

#### Internal routines and practices

Overall, interviewees agreed that there has been little focus on the PAHER at the organizational level.

«We are only reminded to inform the patients, and we have a practice about what you should say at the first consultation» [#7]

In general, the PAEHR has not been very present in daily discussions among colleagues, and the main topic of concern has been the use of the functionality to deny access to information.

### Patient-provider relationship

#### Transparency

Several respondents mentioned that the PAEHR has led to increased transparency for patients and trust towards health professionals.

«I experience that many patients become much more trustful towards me because I am not writing anything behind their back» [#8]

Increased transparency can, in turn, result in a better dialogue with the patient. It was mentioned that, on several occasions, health professionals talk their patients through the content of the record before they leave the consultation to avoid possible misunderstandings. 

While the increased transparency was welcomed by health professionals, there were also some concerns regarding the reach of the information in the records.

«[…] there shouldn’t be any secrecy about the information on you. At the same time, that information should be secured and shouldn’t be spread. There has to be some limits on openness» [15]

Finally, two interviewees mentioned that the PAEHR can contribute to removing the stigma around mental health problems.

«[…] maybe it contributes to create more openness about mental health in society, that accessing and checking your records is normal and not a taboo» [#2]

#### Unsuitability

Most of the interviewees were not worried about patients getting worse or reacting negatively from reading their record. At the same time, some pointed out that maybe they did not treat the most challenging patients. There seemed to be a consensus that the service is mostly unsuitable for patients with a tendency towards suspicion.

*«The situations which I think have been affected concern some patients with emotionally unstable personality disorder, and then sometimes I have experienced things mentioned in the record which created misunderstandings and needed to be clarified afterwards» [#15]*

Some health professionals were worried that such situations can have severe consequences for those vulnerable patients and even cause harm.

«if the patient is so unstable, this kind of information may result in a worsening of symptoms, self-harm, or suicide» [#4]

In general, health professionals tend to present and discuss new diagnoses face-to-face with the patient during consultations, thus avoiding patients to read them online while alone at home. Finally, one interviewee mentioned situations in which patients decided to actively opt out from the possibility to read their record.

#### Relationship with the patient

The PAEHR is considered to have a positive impact on the patient-provider relationship in the vast majority of the situations.

«I think that being more open can be positive for the cooperation with the patient and makes it easier to develop an alliance» [#9]

There are, however, some situations of disagreement in which the service can impact negatively on health professionals’ relationship with their patients. Some interviewees also pointed out that the record as a means of communication between health professionals (e.g. hospitals and GPs) could come in conflict with the relation to the patient.

*«It came from the GP and was added to the record as a note, and I answered that I would discuss it with the patient at the next consultation. But then the patient had already read the record. So when she came to the appointment, she was pretty angry with me and the GP because we had discussed whether or not she was taking her medication» [#10]*

Even if the PAEHR could potentially harm the relationship with the patient, the need to include information which is relevant for the treatment was considered more important than avoiding a possible conflict.

«I have been aware that I shouldn’t omit things I understood or important assessments […], I always include important elements despite there might be reactions» [#4]

Moreover, it is often possible to repair situations of conflict and clarify things with patients who, in turn, can benefit from a learning effect beyond the therapy room.

#### Roles of caregivers, children and third parties

Interviewees pointed out that there is a general focus on writing about caregivers, both children and adults, avoiding details. When writing about third parties information in the record, it is important to be careful and try to make the information unidentifiable.

«[…] third parties should not be identifiable in the record […] you shouldn’t write “molested by stepfather”, right, rather that the patient has experienced it “in the family”» [#5]

Some health professionals were concerned with the increased accessibility to the record due to the PAEHR. For instance, there could be problems in relation to child protection when parents have access to their children’s record. Some interviewees also mentioned problems with adult caregivers, such as negative consequences when the information is willingly shared with partners or when patients are forced by their partners.

«People who live in coercive relationships, maltreated, can be forced to log in» [#12]

The possibility to deny access to information in the record in the aforementioned situations could be used to protect the patient against abusive partners or to protect the child from controlling parents.

#### EHR can be used as legal document

The respondents were well aware that the record was not only being read by the patient, but could potentially also be used as a legal document. As such, health professionals might need to stand accountable for the information they wrote in the record in a trial.

#### Use of PAEHR in treatment

Through the PAEHR patients can follow their treatment and often gain a better understanding. Some mentioned that, by accessing documents such as referrals patients are more informed about their clinical situation and future appointments.

«We use it actively in some treatment courses, if there are some comments to the last notes, […] these can be brought up at the next consultation» [#14]

An active use of the service is also useful to establish a better relationship with the patient, avoid misunderstanding about the treatment or clarifying possible mistakes. The PAEHR can also be used as a way to give homework assignments, thus supporting the treatment and increasing compliance.

«Sometimes I have used it as a tool. As a reminder to the patient where I might add some tasks related to the therapy, diagnostic assessments and such things» [#6]

Some of the respondents expressed a willingness to use the service more actively in patient treatment in the future. However, it was also mentioned that the communication through the record is not the way to go as the use of the PAEHR is so different among patients, and that access to the service could even disturb the treatment.

### Way of writing in the EHR

#### Changes in writing

Overall, there is a common agreement that the quality of the notes has improved over the years. Sseveral interviewees mentioned that they have become more careful about what they write and how they write it, and that the awareness that patients can read their notes might result in using a more objective and understandable language. In specific, many interviewees claimed that journal notes have become shorter, more objective and concise, thus including fewer details.

«A little shorter and more concise notes instead of longer hypotheses and things that you can keep to yourself rather than writing them in the journal» [#6]

Another change that emerged is that the style has become more formal and the syntax has been adapted accordingly.

«I don’t write ‘I’, I write ‘the undersigned» [#16]

At the same time, health professionals make less use of medical terms, and the style has been adapted to be more understandable to the patient.

«I think we were too accustomed to use words no one else knew» [#12]

Still, some mentioned that medical terms cannot be completely avoided as the record is used to communicate with other health professionals.

*«I have used some technical terms where it was needed. This is important when I want to communicate with other specialists. If I need to use some technical terms, I rather explain them to the patient» [#11]*

Finally, health professionals have become more careful in writing information about third parties.

While many interviewees agreed that the content of the records had changed over the years, some pointed out that this was not necessarily due to the introduction of PAEHR specifically, as they always wrote the journal having in mind that the patients had the right to access their records even before the introduction of the service. Over the past years there has also been an increased focus in higher education on how to write journal notes.

#### Changes in workflow

Overall, health professionals did not report that the PAEHR had important consequences for their workflow. Only a few interviewees mentioned that the sequence of actions may have been affected.

«If I have some hypotheses, I try to present them to the patient first, and eventually write them down afterwards» [#11]

#### Consequences for EHR as work tool

Overall, the interviewees did not perceive that the PAEHR had negative consequences for the EHR as a work tool for health professionals. The main challenge is related to setting preliminary diagnoses and assessments, which are part of health professionals’ work documentation, when they know that the patient is able to read them. One interviewee pointed out that the order of information has changed, and health professionals are less in control of when the patient receives information.

*«[…]and then the document is approved in the journal. The patient is supposed to be informed a couple of days later, but meanwhile the patient has already read the diagnostic evaluation and knows that you have set a diagnosis of (inaudible) personality disorder» [#10]*

### Practices to deny access to information

#### Knowledge and use of the functionality

The overall knowledge of the functionality to deny access to information is mixed. Health professionals with more experience with the PAEHR know the functionality well. Overall, the functionality is used seldom by those who use it, only once or just a few times a year. Examples of use of the functionality include psychotic patients who await hospitalization, assessments of chronic suicidality in emotionally unstable patients or patients with personality disorders, and situations involving child protective services.

#### Reflections around the functionality

Those who did not use the functionality seemed to recognize that it could potentially be used in some situations. However, they felt that the threshold for denying access to information is rather high. It was pointed out that the option to deny electronic access to the journal was only to be used in the most severe cases (i.e. risk for endangering the patient’s life or serious damage to the patient’s health).

#### Avoid to write in the EHR

Information is normally not omitted from the journal. This only happens when health professionals are uncertain about the information (e.g. early thoughts of possible diagnoses, theories or hypothesized problems) or for some specific types of information and observations, for instance regarding intelligence testing.

«I don’t want the patient to read their IQ result so I don’t write it down» [#5]

#### Other methods of making information not accessible

Only two interviewees mentioned the possibility for waiting to sign journal notes so that they are not made immediately available to the patient.

«If someone is uncertain about whether the patient would agree with the content, it is possible to wait to sign a document» [#13]

Finally, some health professionals kept a physical folder with some notes.

*«We don’t have […] a parallel journal or a hidden journal. We have a physical folder, but that is more for everyday notes, tests and other documents that have not yet been scanned and entered into the EHR» [#10]*

## Discussion

Mental health professionals have voiced concerns that the transparency of the PAEHR may cause unnecessary worry, confusion, or distress among patients who read their notes without guidance from their clinicians [21, 25], thus damaging the patient-provider relationship [11, 22]. All respondents in the current study were positive towards the transparency provided by the PAEHR and its impact on the patient-provider relationship. This is in line with other studies which reported that health professionals were positive towards the PAEHR both one year [28] and several years after the introduction [32]. A low frequency of situations in which patients were distressed after reading their record online was reported by the interviewees in this study. Previous research indicates that almost all patients feel either better or the same about their providers after receiving access to their record [33]. Overall, the PAEHR does not harm the patient-provider relationship, but it rather has the potential to enhance the provision of patient-centered care [11, 12] and improve trust and satisfaction [10, 33, 34]. Moreover, patients are rarely troubled about the content of the record and that the benefits outweigh the risks in both primary care and specialist care [35].

While confirming that the PAEHR has in general a positive impact on the patient-provider relationship in mental health care, a recent scoping review concluded that the concerns voiced by health professionals about the PAEHR leading to increased documentation workload and possible harm to the patients from reading the notes are not trivial [36]. Results from a previous survey among health professionals in Norway also suggested that the service might not be suitable for the sickest and most vulnerable patients, such as those suffering from schizophrenia, bipolar disorder as well as those with severe depression/suicidality [28]. Similar concerns about patients with severe mental conditions were also identified among some of the participants in the present study. None of the interviewees indicated that they had received specific training in how to write notes for different diagnoses, and such differentiation in documentation practice based on diagnosis is not mandated by the laws and regulations governing health records in Norway. Still, health professionals have some room when deciding what to write in the record, and it is possible that ad hoc training, such as the Open Notes guide to mental health professionals [36], could alleviate some of the concerns. Finally, several providers were under the impression that few patients used the PAEHR service, and the PAEHR was not a central end recurring theme in clinical consultations. The reasons for non-use among patients have not been extensively explored, but a recent qualitative study pointed out that patients may themselves have rationales against using the PAEHR, such as finding it unnecessary, fear provoking and energy demanding [37]. Furthermore, most patients with a psychiatric condition prefer to receive bad news concerning their health during visits and not through the service [38].

In several studies, mental health professionals reported making changes to their note working practices after the introduction of the PAEHR [7, 21, 25, 28, 29]. Health professionals in the present study stated that the documentation practices were subject to changes over the years. However, it was not clear to what extent the changes were attributable to the introduction of PAEHR in 2015. There was a consensus that the quality of the journal notes had improved over the years. Several interviewees pointed out that they have always written their notes keeping in mind that patients might read them, even before the introduction of the service. Interviewees also reported being more concise and avoiding writing hypotheses which might create confusion in patients. A possible paradox was that health professionals reported using fewer medical terms, but also a more formal syntax. The providers reported that their workflow was not severely affected by the PAEHR. The low impact on workflow is supported by a study of oncology health professionals, reporting that although there were some consequences for documentation practices, specifically that they are more restrictive in what they write in the notes, the overall adverse impact of PAEHR did not match the concerns prior to implementation [39]. Future research should study in detail changes in documentation practices by analysing the content of the EHR.

Results from previous studies show that several mental health professionals were worried that information in the EHR might harm the patient or their relationship with the patient and discussed with a colleague whether to deny access [28, 29]. Interestingly, the functionality to deny patients access to information was seldom used by the interviewees of this study, and some reported that they had never used it. It is unlikely that the reason for the limited use is due to the technical inability to do so, as the procedure was not deemed to be complicated. A patient may be denied access if this is absolutely necessary to avoid endangering the patient’s life, serious damage to the patient’s health, or harm to persons close to the patient. The interviewees’ impression was that the threshold for denying access is high and that the functionality should only be used in severe cases. As a consequence, the limited use of this functionality can be considered to be intentional. Some health professionals mentioned that sometimes they delay the final approval of their notes so that they are not made immediately available in the PAEHR. This practice is also mentioned as a possibility in the guidelines. Previous research indicated a higher prevalence of information storing outside the EHR, so called “hidden” or “shadow” journal, in psychiatry compared to somatic healthcare [29]. However, according to the respondents of the present study, such a practice did not occur in their outpatient clinics.

### Limitations

In the present study, 16 health professionals were interviewed from two locations within the same institution. Towards the end of the analyses, each new transcript yielded a low number of new codes and themes, indicating that the data material had achieved inductive thematic saturation [40]. It is therefore likely that the results from this study present a true image of the providers’ experiences from using the PAEHR at the two participating outpatient clinics. Although there are similarities in organization, staff composition and patient population among outpatient clinics within the public healthcare system in Norway, it is also likely that procedures and internal training might differ across different institutions. As a consequence, the findings from this study might have overexposed or underexposed particular aspects of mental health professionals’ experiences related to the PAEHR. Caution should be applied when generalizing the results and drawing hypotheses of health professionals’ impressions on the PAEHR, especially in contexts which are very different from the investigated locations, such as inpatient clinics.

## Conclusions

The PAEHR implemented in Norway was seen as a tool to increase transparency and improve patient-provider relationship. The interviewees confirmed that the PAEHR could have negative consequences only in limited situations, such as for patients with severe mental conditions, for child protection when parents access their children’s records or for patients with abusive partners. The functionality to deny access to the journal to protect the patient in such situations was used rarely. A more common practice for making information not immediately available to the patient was to delay the final approval of their notes. The concerns voiced by mental health professionals regarding the impact of the PAEHR on the patient-provider relationship and practices to deny access to information were not supported by the results of this study. Health professionals in the present study stated that the documentation practices were subject to changes over the years. However it was not clear to what extent the changes were attributable to the introduction of the PAEHR. Most of the interviewees write their notes keeping in mind that patients might read them, and they try to avoid unclear language, information about third parties, and hypotheses that might create confusion. Future research should explore changes in documentation practices by analysing the content of the EHR.

## Data Availability

The data supporting the results of this study are available upon request to the corresponding author (Paolo Zanaboni, paolo.zanaboni@ehealthresearch.no).

## Abbreviations

EHR: Electronic health record
PAEHR: Patient accessible electronic health records
